# Efficacy and Cost Effectiveness of the Acupuncture Treatment Using a New Skin Stimulus Tool Called *M*-Test Which Is a Measure Based on Symptoms Accompanied with Body Movements: A Pragmatic RCT Targeting Hemodialysis Patients

**DOI:** 10.1155/2015/802846

**Published:** 2015-05-03

**Authors:** Shuji Ono, Yoshito Mukaino

**Affiliations:** ^1^Department of Oriental Medicine of Fukuoka University Hospital, 7-45-1 Nanakuma, Jonan-ku, Fukuoka 814-0180, Japan; ^2^Faculty of Sports and Health Science, Fukuoka University, 8-19-1 Nanakuma, Jonan-ku, Fukuoka 814-0180, Japan

## Abstract

*M*-Test can simultaneously reduce hemodialysis patients' diverse symptoms. Its diagnosis and treatment are based on simple movements that can be performed by anyone and allow determining which meridians have problems by analyzing symptoms accompanied with movement. It also enables to conduct a safe and effective treatment with use of microcorn which is a noninvasive treatment tool. This time we conducted microcorn intervention on hemodialysis patients based on diagnosis of *M*-Test. As a result, almost all of the dialysis patients' complaints have been relieved while the score of HR-QOL increased. According to our calculation of cost effectiveness, it confirmed that it is very cost-effective.

## 1. Introduction

Although every hemodialysis patient has multiple symptoms [1], many previous studies on the efficacy of acupuncture treatment for hemodialysis patients were focused on their typical symptoms individually such as itchiness, fatigue, insomnia, and pain [2, 3]. However, a hemodialysis patient has a wide range of nonmotor symptoms such as itchiness, dizziness, and palpitations in addition to orthopedic pain including back pain. Acupuncture treatment can simultaneously reduce a wide range of dialysis patients' symptoms; however, there have been little studies discussing the efficacy of acupuncture treatment focusing on all symptoms that a single patient suffers.

With* M*-Test [4, 5], our new acupuncture therapeutic method introduced in 1995, diverse symptoms of dialysis patients can be simultaneously relieved.* M*-Test is a treatment approach to diagnose and treat patients by using a measure based on symptoms accompanied by body movements such as pain and dizziness. Its basic movements consist of simple movements that anyone can perform. By analyzing symptoms accompanied by movements, we can easily determine which meridians have problems and how they should be restored and what kind of treatments are required.* M*-Test is simple and safe and treat patients' complaints effectively and quickly [6].

By simulating acupuncture points that are led by this method, many case studies show that a patient's various symptoms get simultaneously relieved when the body movement gets improved. Multiple medicines are required to treat diverse symptoms of dialysis patients based on Western medicine. It is of a low cost and has a significant economic benefit to be able treat patients without medicine.

In addition, needles have been mainly used as a stimulating tool in discussing efficacy of acupuncture treatment for dialysis patients. It is a safe treatment approach if conducted by trained acupuncturist; however, there are risks of increased susceptibility to infection due to their lower immunity as well as hemorrhage from use of an anticoagulant. Therefore it is desirable to use a less-invasive or noninvasive stimulation approach.

In the current clinical study for hemodialysis patients, we used a noninvasive skin stimulating tool which was developed in 2006. Type II is a brush (or microcorn) which has approximately 400 microspikes that are regularly aligned on a disc in diameter of 11 mm. It is not inserted but only lightly stimulates the skin surface with microspikes. For this reason, there is no danger of bleeding and infection and it is pain-free for patients. Based on our experiences, it has equivalent or even more effects to use it as a stimulating tool during acupuncture treatment for various symptoms. In animal experiments, it has been reported by Hotta et al. that the light mechanical stimulation by the microcorn can induce a strong analgesic effect on autonomic reflex [7]. Efficacy of microcorn is becoming clear in the fundamental medicine.

If we can prove effectiveness of the skin stimulating tool for acupuncture points led by the newly developed symptoms, we believe it will open up acupuncture's new possibilities not only for hemodialysis patients' symptoms but also for various diseases in different areas.

Therefore, we conducted a pragmatic randomized controlled trial (P-RCT) for hemodialysis patients using the acupuncture point selecting method based on* M*-test which is measured by symptoms accompanied by body movements and newly developed microcorn symptoms in order to discuss its efficacy and cost-effectiveness of the treatment.

## 2. Subjects and Methods

### 2.1. Subjects

We targeted 47 patients who understood and agreed to the study's purpose and content out of 100 hemodialysis outpatients at hemodialysis facilities in Kagawa Prefecture. Primary diseases which led to the introduction of hemodialysis included 16 chronic glomerulonephritis, 17 diabetic nephropathy, 4 nephrosclerosis, 1 chronic pyelonephritis, 1 ANCA-related nephritis, 1 IgA nephritis, 1 membranous nephropathy, 1 secondary hyperparathyroidism, and 5 unknown causes.

### 2.2. Condition of Maintenance Hemodialysis

We made a comparison on regular monthly blood test results for patients with maintenance hemodialysis in order to understand effectiveness of hemodialysis, abnormal bone metabolism, anemia, and self-management. Measurement of blood test included erythrocyte, leukocyte, hematocrit value, hemoglobin, urea nitrogen, creatinine, calcium, inorganic phosphorus, total protein, and albumin.

### 2.3. Study Design

Subjects who receive maintenance hemodialysis were randomly allocated to 2 groups: one with acupuncture treatment (acupuncture group) and one without it (traditional treatment group). We allocated subjects by a draw using envelopes. Both groups continued dialyzing before intervention for a hemodialysis.

This research was conducted for 21 weeks and the pretreatment was set as treatment phase 0. For the group with acupuncture, treatment was given once a week and 8 times in total. We defined a week after the first treatment as treatment phase 1, a week after the second treatment as treatment phase 2, and then phases 3, 4, 5, 6, 7, and 8 subsequently on a weekly basis. We also defined 4 weeks after treatment phase 8 as follow-up phase 4 and after 12 weeks as follow-up phase 12. For the traditional treatment group, only hemodialysis was given with the same schedule of observation criteria. Symptoms and EQ-5D were researched by survey sheet 5 times in total at treatment phase 0, treatment phase 4, treatment phase 8, follow-up phase 4, and follow-up phase 12. Observation criteria will be explained more in detail in a later chapter. Each survey sheet is filled directly by a patient and collected before starting hemodialysis. Blood test was performed on monthly basis.

### 2.4. Treatment Methods

*M*-Test was employed for acupuncture treatment for the acupuncture group.* M*-Test is a diagnostic treatment system that has a measure for symptoms accompanied by body movement. This method allows determining problematic meridians by analyzing symptoms associated with movement. By performing movements classified into 30 items and facilitating positive findings induced by a movement such as pain and tension as Numerical Rating Scale (NRS), this method evaluates 11 degrees of conditions between 0 which is a state free of pain and tension to 10 which is a state with the strongest pain and tension. It allows us to easily determine how meridians should be restored and what kind of treatments is required by summarizing the result.* M*-Test is simple and safe and treats patients' complaints effectively and quickly. As the body movement is improved, not only locomotory symptoms but also various symptoms including aplanetic ones can be simultaneously improved. Treatment procedure in detail conformed to a principle of* M*-Test. First, therapist measures patient's* M*-Test finding. 30 items of* M*-Test finding consist of movement by which the anterior, the posterior and the lateral-medial are extended. Movement is classified by the upper part of body and the lower part of the body and it is classified into 6 blocks (anterior (A block), posterior (B block), and lateral-medial (C block) upper body; anterior (D block), posterior (E block), and lateral-medial (F block) lower body). Therapist estimates patient's movement in NRS and this is* M*-Test finding.

The occasion with the finding is regarded as abnormality of the meridian distributed over the block. In other words, when there is abnormality in an A block, it is a problem of a lung and large intestine meridians; in a B block, it is a problem of a heart and small intestine meridians; in a C block, it is a problem of a pericardium and triple energizer meridians; in a D block, it is a problem of a spleen and stomach meridians; in a E block, it is a problem of a kidney and bladder meridians; and in a F block, it is a problem of a liver and gallbladder meridians. A basic acupuncture point is set in* M*-Test. It is LU5 LU9, LI2, and LI11 in A block, HT7, HT9, SI3, and SI8 in B block, PC7, PC9, TE3, and TE10 in C block, SP2, SP5, ST41, and ST45 in D block, KI1, KI7, BL65, and BL67 in E block, and LR2, LR8, GB38, and GB43 in F block. Next, therapist treats a patient. Therapist touches patient's basic acupuncture point, and a patient does the movement with positive movement of* M*-Test finding. Therapist chooses the most effective acupuncture point for a patient and treats it with a treatment tool. Therapist obeys the following rules. (1) A therapist checks all* M*-Test findings. (2) It is treated from a lower body. (3) It is treated from the highest score of* M*-Test finding.

When a basic acupuncture point shows the enough effect, a medical person treats the next point. If the effect by the basic treatment point is insufficient or it is ineffective, therapist treats muscle on the meridian that runs each block. This is repeated until all* M*-Test findings decrease. When a* M*-Test finding was not improved, the next step was prepared, but a skill until this stage was used by this study.

### 2.5. Treatment Tool

Microcorn (Somacept type III and Somareson type III), noninvasive simulating tools manufactured by Tokyo Resin Corp, was used in this study. Somareson type III is a small disc of 4 mm in diameter and attached to a tape. It is made of elastomer and has about 40 microspikes in height of 0.03 mm on the stimulating surface. The microspikes are regularly aligned at interval of 0.4 mm and lightly stimulate skin surface with a fixed pressure by attaching to the skin. Although it is same form and size as Somacept type III, its material is made of hard plastic and its microspike has a different shape. It is 0.15 mm in height while its grid interval is 0.45 mm.

### 2.6. Patient's Symptoms

Multiple-answer questionnaire of 40 criteria was created with use of reference documents to study patients' symptoms [8]. We selected 20 criteria that have the highest symptom prevalence. Symptoms shown from the highest occurrence are as follows: headache, blurred vision, difficulty in hearing, dizziness, ear buzzing, palpitation, depression, constipation, cervical pain, stiff shoulders, back pain, knee pain, leg cramp, lower limb pain, numbness in upper limb, numbness in lower limb, itchiness, difficulty in sleeping, edema, and listlessness. Each symptom was evaluated by its degree with Visual Analogue Scale (VAS) [9].

### 2.7. EQ-5D and Utility

QOL (quality of life) score (utility) was calculated by using EuroQol5-Dimension (EQ-5D) questionnaire [10, 11]. EQ-5D is a questionnaire used to evaluate health related QOL (HR-QOL) as a preference-based measure developed in UK. It consisted of five-dimensional approach and visual evaluation. The currently collected responses from the five-dimensional approach were computed with Tarrif Value set or so-called conversion for utility that converted death as 0 and perfect health as 1 which is one-dimensional QOL specified by an interval scale. Utility collected from the five-dimensional approach was used in this study for cost analysis.

### 2.8. Incremental Cost-Effectiveness Ratio (ICER)

As an effective index for cost effectiveness analysis, utility calculated by survey sheets and survived period under the applicable conditions was multiplied while the index called quality-adjusted life years (QALY) was used to consider both the quality and the extension of life [12]. In this research, we calculated ICER, the amount of money required to increase QALY for a year, and used utility which was collected from the results of EQ-5D. We calculated ICER as the cost of* M*-test per a patient in the acupuncture group and that of medicines actually prescribed as direct costs:

(1) ICER  Yen/QALY =Materials  used  for  acupuncture  treatment  ·QALY  for  treatment  phase  8  less  QALY   right  before  introduction−1.

### 2.9. Statistical Processing

We conducted two-way repeated measures analysis of variance to evaluate the groups (two standards: acupuncture group and traditional treatment group) with VAS and utility, as well as 2 factors of each group's timeline. Significance level was set below 5% while SPSS15.0 was used for statistical processing.

## 3. Results

### 3.1. Characteristics of Patients

We clarified patients in each group for their height, dry weight (DW), age, average years of dialysis, standard deviation, and primary diseases. There was not a significant characteristic difference in those patients (Table 1).

During the study, one patient dropped out of acupuncture group due to hospitalization to another hospital. Six patients dropped out from the traditional treatment group for reasons specified as follows. One patient deceased, 4 patients were hospitalized to other hospitals, and 1 patient refused to respond to the questionnaire.

### 3.2. States of Maintenance Hemodialysis

Blood test results were compared by each criterion (Table 2). There was no significant difference for all criteria between the two groups.

### 3.3. *M*-Test Finding

**Figure 1:**
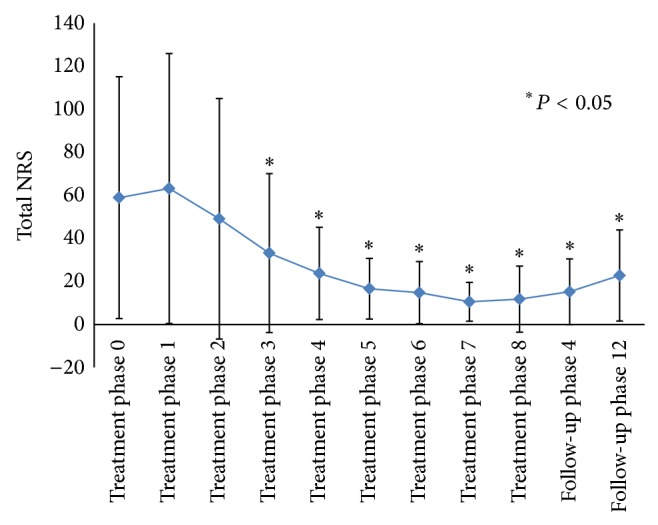
Total NRS of* M*-test finding.

To achieve findings for* M*-Test, we compared the total points of all movements' NRS collected at each pretreatment (Figure 1). Comparing from treatment phase 0, total NRS went down significantly after treatment phase 3.

### 3.4. Patients' Symptoms

The results from the questionnaire are shown in the Table 3. There was no criterion with a significant difference at treatment phase 0 between the two groups. During the measurement period, 12 VAS of symptoms were significantly decreased for acupuncture group compared from treatment phase 0 as follows: headache, blurred vision, dizziness, ear buzzing, cervical pain, stiff shoulders, back pain, lower limb pain, numbness in lower limb, numbness in upper limb, itchiness, and difficulty in sleeping. For the traditional treatment group, 3 symptoms were decreased as follows: difficulty in hearing, stiff shoulders, and difficulty in sleeping. During the measurement period, variances were confirmed between the two groups for the 12 VAS of symptoms as follows: blurred vision, dizziness, palpitation, depression, constipation, cervical pain, stiff shoulders, leg cramp, numbness in lower limb, numbness in upper limb, itchiness, and difficulty in sleeping. In all symptoms, the acupuncture group showed significantly low values. In addition, most of symptoms decreased their value from treatment phase 0 to 8 and increased from treatment phase 8 to follow-up phase 12.

### 3.5. Utility

Utility collected by EQ-5D is shown in Table 4. Utility of those in acupuncture group increased significantly at treatment phase 8 while utility of those in traditional treatment group confirmed no significant difference. In addition, the acupuncture group showed significantly high points at treatment phase 8.

### 3.6. Costs and ICER

The amount of microcorns spent per patient was 4,328 Yen (SD = 778 Yen) at average. Quantity of microcorn used in a single* M*-Test was 4 at least and 20 at most with an average of 10.8 (SD = 1.94). The cost per a single* M*-Test was 540 Yen. ICER calculated based on the utility collected by EQ-5D questionnaire was 980,998 Yen/QALY.

## 4. Discussion

### 4.1. Patients' Symptoms

Every hemodialysis patient in this clinical study has multiple symptoms. For example, they have a wide range of nonmotor symptoms such as itchiness, dizziness, and palpitations in addition to orthopedic pain including back pain. It is a widely known fact that these symptoms limit activities and decrease QOL due to physical and mental loads. Multiple medicines are required to treat them with traditional western medicine and can be expensive.

After 8 weeks of acupuncture treatment in this study for the acupuncture group, 12 symptoms out of 20 were significantly improved. Six out of 12 were locomotory disorders including cervical pain, stiff shoulders, back pain, lower limb pain, numbness in lower limb, and numbness in upper limb. The remaining 6 were other types of disorders included headache, blurred vision, dizziness, ear buzzing, itchiness, and difficulty in sleeping. Since* M*-Test treats movement, it is obvious that locomotory symptoms get improved. In addition it confirmed that it is also effective to improve symptoms other than locomotory symptoms. This result indicates that multiple symptoms can be simultaneously treated by focusing on treatment on abnormal movements. Symptoms improved by the traditional treatment group such as blurred vision, difficulty hearing, and stiff shoulders were improved due to seasonal variations and the effect of traditional dialysis treatments while the 2 symptoms including blurred vision and stiff shoulders were believed to reduce symptoms by adding* M*-Test since these showed significantly low points in acupuncture treatment group apart from seasonal variations. We believe addition of* M*-Test to the traditional dialysis treatments enabled to effectively treat other symptoms such as dizziness, palpitation, depression, constipation, numbness in lower limb, numbness in upper limb, itchiness, and difficulty in sleeping. Judging from these said reasons and the status quo that dialysis patients with multiple disorders are prescribed a medicine per symptom, we believe* M*-Test treatment can simultaneously treat multiple symptoms and can be an effective treatment by improving body movements. Previous clinical observations also indicate that dialysis patients' symptoms are deeply related to aggravation of body movements. It is assumed that one of causes for a development or aggravation of a symptom is related to worsened body movement.

### 4.2. Utility and QOL

In order to examine the influence for EQ-5D and QOL, we researched utility that is an index of HR-QOL for 5 times in total including treatment phase 0, treatment phase 4, treatment phase 8, follow-up phase 4, and follow-up phase 12. Collected QOL was significantly high for acupuncture group at treatment phase 8 (*P* < 0.05). QOL is the index of HR-QOL and if it has increased, it translates as improvement of the dialysis patient's QOL.* M*-Test treatment for 8 weeks has reduced dialysis patients' symptoms and it is believed that the QOL has improved as a result. We did not confirm a change in utility at EQ-5D upon treatment phase 4 when the 4th treatment out of 8 was completed which indicated that it might require a certain period of treatment to expect effectiveness of the adequate degree to increase QOL.

### 4.3. ICER

ICER calculable from utility is the differences of medical fees less than QALY before and after the interventions and indicates the cost required to prolong 1QALY. In this case, it is equivalent of the medical fees including the cost of microcorns required to extend a year of dialysis patient's QALY. The cost of microcorns required for this study as well as dialysis patient's ICER calculated from the varied traditional dialysis treatment costs was 980,998 Yen per QALY. Okusa has reported the affordable cost per QALY for the Japanese society as 6 million Yen [13]. These 6 million Yen are reviewed as a maximum amount for cost-effectiveness upon discussing a new medical practice and it is concluded to be cost-effective if it is below this amount.

### 4.4. *M*-Test

*M*-Test treatment differs from traditional acupuncture treatment. It is very simple and safe, which allows quick judging and getting results effectively. There are a lot of choices in a check, a remedy, a skill, and a tool for conventional acupuncture and a variable of the factor by which public welfare does that is too big for the check and treatment, and it is difficult to indicate reproducibility and objectivity.

But the* M*-Test is also similar to the manual muscle testing a doctor and a physical therapist use, so it is easy for a therapist to understand. The therapeutic effect does not influence skill of a therapist, like a sphygmoscopy or tongue diagnosis. Reproducibility is high because a procedure from a check to treatment is decided even if an unskillful therapist treats a patient in* M*-Test. We can tell the self-care for which* M*-Test was utilized to a patient when a patient understood that various symptoms decreased by improving* M*-test finding. Since patient's reaction can be confirmed on sites, it allows medical practitioners to learn effective points for treatment and improve their clinical techniques.* M*-Test requires a short treatment which will hardly be a burden for patients and is very simple and easy to introduce to the traditional dialysis treatments. In addition, patients become more conscious of their own health management as they can confirm effectiveness with their body movement. Once they understand their own characteristics, they can conduct self-care by stretching since the movements of* M*-Test are in form of stretching at the same time. In considering characteristics of dialysis treatment, it is very important for patients to be able to perform self-care. It is also promising to reduce medical costs.

It is believed that* M*-Test treatment is cost-effective and effective to reduce patients' symptoms and maintain and improve QOL.

### 4.5. Microcorns

Emergence of microcorn has opened up possibilities for acupuncture treatment to be an applicable approach for other medical license holders in the future such as nurses and physiotherapists while acupuncture treatment is traditionally performed by limited practitioners such as doctors and acupuncturists. In addition, there are possibilities to develop it as a methodology that can be performed by patients as self-care. We have been advising patients to use microcorns as self-care and are confident it will be a very effective self-care method.

## 5. Summary

In this study, we researched dialysis patients' symptoms and effects on QOL to evaluate its economic performance. The result indicated that an introduction of acupuncture treatment to dialysis treatment is cost-effective and beneficial to reduce dialysis patients' symptoms and improve QOL. In particular, use of* M*-Test allows safe and prompt evaluation, as well as delivery of quantified effects. Use of microcorn also expands places and situations where nonacupuncturists can practice acupuncture treatment which can be a very useful self-care method.

## Figures and Tables

**Table 1:** Demographic characteristics and laboratory date at time of study recruitment.

	Acupuncture group	Traditional treatment group
Number of patients (*n*)	24	23
Age (years)	70.0 ± 9.6	67.3 ± 13.0
Height (cm)	155.6 ± 10.8	158.9 ± 9.5
Dry weight (kg)	50.0 ± 9.4	53.2 ± 11.7
Time on hemodialysis (years)	10.3 ± 8.2	6.5 ± 6.8
Primary diseases		
Chronic glomerulonephritis	10	6
Diabetic nephropathy	6	11
Nephrosclerosis	2	2
Chronic pyelonephritis	1	0
ANCA-related nephritis	1	0
IgA nephritis	1	0
Membranous nephropathy	1	0
Secondary hyperparathyroidism	1	0
Unknown cause	1	4

No significant differences between the two groups.

**Table 2:** Blood test results were compared by each criterion.

Blood test	Group	Treatment phase 0	Treatment phase 4	Treatment phase 8	Follow-up phase 4	Follow-up phase 12
Leukocyte (10^2^/*μ*L)	Acupuncture group *n* = 23	366.7 ± 40.7	357.7 ± 40.5	352.6 ± 37.3	365.6 ± 51.4	365.2 ± 53.8
	Traditional treatment group *n* = 17	371.1 ± 65.2	364.5 ± 78.2	340.6 ± 55.7	362.7 ± 58.2	364.2 ± 48.7
Leukocyte (10^2^/*μ*L)	Acupuncture group *n* = 23	51.8 ± 14.9	51.7 ± 18.6	51.8 ± 18.4	54.3 ± 19.4	54.6 ± 19.5
	Traditional treatment group *n* = 17	63.6 ± 22.5	62.8 ± 22.5	60.1 ± 23.4	63.6 ± 23.8	66.4 ± 29.7
Hematocrit value (%)	Acupuncture group *n* = 23	35.2 ± 3.0	34.8 ± 3.7	35.0 ± 3.2	35.4 ± 4.2	35.3 ± 4.4
	Traditional treatment group *n* = 17	36.5 ± 4.5	36.7 ± 6.0	34.3 ± 4.3	35.9 ± 4.0	36.3 ± 2.9
Hemoglobin (g/dL)	Acupuncture group *n* = 23	11.7 ± 1.1	11.6 ± 1.2	11.7 ± 1.0	11.9 ± 1.4	11.7 ± 1.5
	Traditional treatment group *n* = 17	12.1 ± 1.4	12.2 ± 1.9	11.6 ± 1.4	12.1 ± 1.3	12.2 ± 1.0
Urea nitrogen (mg/dL)	Acupuncture group *n* = 23	22.7 ± 5.9	22.6 ± 7.3	19.1 ± 5.0	18.7 ± 5.4	20.0 ± 5.7
	Traditional treatment group *n* = 17	214.4 ± 8.2	21.7 ± 6.0	20.6 ± 6.5	22.1 ± 8.2	22.4 ± 8.1
Creatinine (mg/dL)	Acupuncture group *n* = 23	4.1 ± 1.1	4.2 ± 1.1	4.0 ± 0.9	4.0 ± 1.0	4.8 ± 3.3
	Traditional treatment group *n* = 17	4.5 ± 1.0	4.4 ± 1.1	4.2 ± 1.0	4.7 ± 2.2	4.8 ± 2.2
Calcium (mg/dL)	Acupuncture group *n* = 23	9.5 ± 0.6	9.7 ± 0.5	9.5 ± 0.4	9.4 ± 0.5	9.4 ± 0.6
	Traditional treatment group *n* = 17	9.7 ± 0.6	9.8 ± 0.8	9.8 ± 0.7	9.8 ± 0.5	9.6 ± 0.5
Inorganic phosphorus (mg/dL)	Acupuncture group *n* = 23	2.4 ± 0.5	2.5 ± 0.6	2.3 ± 0.5	2.3 ± 0.4	2.2 ± 0.4
	Traditional treatment group *n* = 17	2.6 ± 0.6	2.4 ± 0.5	2.3 ± 0.6	2.5 ± 0.9	2.4 ± 0.7
Total protein (g/dL)	Acupuncture group *n* = 23	6.1 ± 0.6	8.5 ± 11.4	6.1 ± 0.5	5.9 ± 0.5	6.0 ± 0.5
	Traditional treatment group *n* = 17	6.5 ± 0.4	6.6 ± 0.3	6.5 ± 0.2	6.3 ± 0.3	6.6 ± 0.4
Albumen (*μ*mol/L)	Acupuncture group *n* = 23	3.6 ± 0.2	3.6 ± 0.2	3.5 ± 0.2	3.6 ± 0.2	3.5 ± 0.3
	Traditional treatment group *n* = 17	3.7 ± 0.2	3.7 ± 0.2	3.6 ± 0.1	3.6 ± 0.1	3.7 ± 0.2

No significant difference between the two groups.

**Table 3:** The change in patient's symptoms in each phase.

Symptom	Group	Treatment phase 0	Treatment phase 4	Treatment phase 8	Follow-up phase 4	Follow-up phase 12
Headache	Acupuncture group *n* = 23	17.1 ± 26.1	10.7 ± 15.3	6.2 ± 13.5^#^	8.5 ± 20.0^#^	8.2 ± 13.8
	Traditional treatment group *n* = 17	8.6 ± 16.3	11.6 ± 20.1	9.9 ± 17.7	8.5 ± 14.3	15.6 ± 26.9
Blurred vision	Acupuncture group *n* = 23	33.4 ± 32.7	17.0 ± 22.2^#^	12.7 ± 20.9^#∗^	13.5 ± 17.9^#∗^	19.4 ± 20.9^#∗^
	Traditional treatment group *n* = 17	34.5 ± 36.3	33.4 ± 30.4	43.7 ± 37.1	33.9 ± 31.5	41.6 ± 30.7
Difficulty in hearing	Acupuncture group *n* = 23	17.8 ± 24.8	17.7 ± 22.7	13.0 ± 22.8	12.4 ± 18.4	18.6 ± 28.6
	Traditional treatment group *n* = 17	20.6 ± 28.9	26.4 ± 31.5	25.6 ± 34.1	29.3 ± 34.6	33.2 ± 36.1^#^
Dizziness	Acupuncture group *n* = 23	13.0 ± 21.4	10.2 ± 17.7	1.4 ± 6.3^#∗^	2.8 ± 7.4^#^	7.9 ± 13.9
	Traditional treatment group *n* = 17	9.4 ± 16.4	14.7 ± 22.9	15.8 ± 23.3	9.1 ± 14.5	8.1 ± 36.1
Ear buzzing	Acupuncture group *n* = 23	17.9 ± 27.2	13.5 ± 23.7	8.0 ± 13.2	8.0 ± 14.7^#^	10.7 ± 19.5
	Traditional treatment group *n* = 17	21.1 ± 30.2	16.0 ± 23.3	21.7 ± 34.3	16.6 ± 28.4	24.3 ± 31.5
Palpitation	Acupuncture group *n* = 23	16.6 ± 27.8	14.0 ± 19.5	6.5 ± 17.2^*^	11.1 ± 23.5	15.3 ± 20.8
	Traditional treatment group *n* = 17	12.2 ± 25.0	18.4 ± 21.1	21.4 ± 27.7	11.4 ± 21.2	16.8 ± 24.4
Depression	Acupuncture group *n* = 23	15.4 ± 29.1	7.8 ± 15.7^*^	6.4 ± 19.3^*^	4.4 ± 8.5^*^	9.7 ± 20.3
	Traditional treatment group *n* = 17	19.3 ± 30.5	24.7 ± 30.7	22.4 ± 30.2	26.8 ± 32.4	21.9 ± 31.6
Constipation	Acupuncture group *n* = 23	25.0 ± 35.1	18.5 ± 30.3	13.7 ± 23.0^*^	7.4 ± 11.7^*^	15.8 ± 25.7^*^
	Traditional treatment group *n* = 17	45.4 ± 41.0	35.6 ± 34.1	31.4 ± 32.9	43.8 ± 39.0	41.4 ± 40.0
Cervical pain	Acupuncture group *n* = 23	37.7 ± 39.1	25.3 ± 29.7^#^	14.2 ± 21.9^#∗^	23.1 ± 24.3^#^	25.1 ± 27.7
	Traditional treatment group *n* = 17	16.0 ± 24.5	25.2 ± 24.9	34.5 ± 33.6	29.5 ± 35.4	30.2 ± 33.5
Stiff shoulders	Acupuncture group *n* = 23	29.9 ± 28.6	34.5 ± 25.6	12.5 ± 21.6^#∗^	21.3 ± 25.2^#^	29.9 ± 28.6^#^
	Traditional treatment group *n* = 17	25.1 ± 27.1	25.9 ± 26.2	37.2 ± 34.2	39.1 ± 39.3^#^	35.5 ± 33.9
Back pain	Acupuncture group *n* = 23	38.5 ± 33.7	27.3 ± 29.6	9.3 ± 18.1^#^	20.7 ± 31.2^#^	33.8 ± 31.8
	Traditional treatment group *n* = 17	25.5 ± 22.8	22.1 ± 27.4	23.0 ± 27.1	28.1 ± 29.9	27.9 ± 26.4
Knee pain	Acupuncture group *n* = 23	26.0 ± 32.1	24.8 ± 28.2	15.7 ± 25.8	20.8 ± 31.5	29.8 ± 31.4
	Traditional treatment group *n* = 17	23.1 ± 31.9	23.8 ± 33.4	15.8 ± 27.5	21.6 ± 33.1	24.3 ± 32.1
Leg cramp	Acupuncture group *n* = 23	20.5 ± 29.1	21.4 ± 29.5	12.1 ± 17.8^*^	15.1 ± 30.0	13.7 ± 24.5^*^
	Traditional treatment group *n* = 17	40.8 ± 37.8	38.2 ± 25.9	33.6 ± 33.1	33.2 ± 32.1	34.7 ± 35.8
Lower limb pain	Acupuncture group *n* = 23	29.4 ± 36.4	29.4 ± 30.1	17.1 ± 23.3^#^	16.7 ± 27.2	23.2 ± 32.7
	Traditional treatment group *n* = 17	21.5 ± 33.9	28.5 ± 35.8	25.5 ± 36.3	27.2 ± 35.5	26.6 ± 34.3
Numbness in upper limb	Acupuncture group *n* = 23	18.9 ± 30.4	12.5 ± 34.9^*^	4.0 ± 29.5^#∗^	9.0 ± 29.0^#^	9.5 ± 26.1
	Traditional treatment group *n* = 17	29.6 ± 40.3	21.0 ± 37.9	11.6 ± 38.6	23.2 ± 40.5	20.1 ± 38.0
Numbness in lower limb	Acupuncture group *n* = 23	21.9 ± 34.9	16.0 ± 25.3^*^	9.8 ± 23.1^*^	11.0 ± 26.2^#∗^	12.6 ± 24.2
	Traditional treatment group *n* = 17	32.5 ± 40.6	39.1 ± 40.7	34.9 ± 33.9	33.0 ± 38.5	29.8 ± 37.5
Itchiness	Acupuncture group *n* = 23	38.7 ± 40.7	29.3 ± 31.5^#^	18.6 ± 29.7^#∗^	18.6 ± 27.7^#^	22.7 ± 28.1^#^
	Traditional treatment group *n* = 17	33.2 ± 35.5	33.3 ± 31.2	43.9 ± 37.2	35.7 ± 39.7	40.4 ± 42.3
Difficulty in sleeping	Acupuncture group *n* = 23	34.8 ± 36.9	28.2 ± 31.2^*^	12.8 ± 22.5^#∗^	12.2 ± 21.4^#∗^	19.6 ± 26.9^#^
	Traditional treatment group *n* = 17	39.3 ± 40.4	54.5 ± 38.0^#^	43.9 ± 37.2	35.7 ± 39.7	40.4 ± 42.3
Edema	Acupuncture group *n* = 23	13.9 ± 27.0	10.3 ± 24.8	8.8 ± 19.8	9.4 ± 22.4	14.0 ± 29.5
	Traditional treatment group *n* = 17	16.1 ± 27.6	17.5 ± 26.1	14.2 ± 26.2	14.6 ± 21.2	18.2 ± 32.9
Listlessness	Acupuncture group *n* = 23	31.7 ± 33.8	26.8 ± 25.6	20.6 ± 29.8	19.9 ± 23.7	29.0 ± 31.3
	Traditional treatment group *n* = 17	41.3 ± 36.4	42.9 ± 32.6	36.4 ± 29.3	38.6 ± 35.5	38.4 ± 33.1

^*^ *P* < 0.05, compared to traditional treatment group.

^#^ *P* < 0.05 compared to the phase 0 in the group.

**Table 4:** The change in utility in each phase.

	Treatment phase 0	Treatment phase 4	Treatment phase 8	Follow-up phase 4	Follow-up phase 12
Acupuncture group *n* = 23	0.66 ± 0.15	0.65 ± 0.18	0.76 ± 0.17^#∗^	0.71 ± 0.16	0.66 ± 0.20
Traditional treatment group *n* = 17	0.64 ± 0.18	0.63 ± 0.20	0.64 ± 0.18	0.63 ± 0.19	0.64 ± 0.21

^*^ *P* < 0.05, compared to traditional treatment group.

^#^ *P* < 0.05 compared to the phase 0 in the group.
